# A Behavior-Based Strategy for Single and Multi-Robot Autonomous Exploration

**DOI:** 10.3390/s120912772

**Published:** 2012-09-18

**Authors:** Jesus S. Cepeda, Luiz Chaimowicz, Rogelio Soto, José L. Gordillo, Edén A. Alanís-Reyes, Luis C. Carrillo-Arce

**Affiliations:** 1 Center for Robotics and Intelligent Systems, Tecnológico de Monterrey, Monterrey 64849, Mexico; E-Mails: rsoto@itesm.mx (R.S.); jlgordillo@itesm.mx (J.L.G.); edenalanis@gmail.com (E.A.A.-R.); lc.carrilloarce@gmail.com (L.C.C.-A.); 2 Computer Science Department, Universidade Federal de Minas Gerais, Belo Horizonte, 31270-901, Brazil; E-Mail: chaimo@dcc.ufmg.br

**Keywords:** multi-robot autonomous navigation and exploration, coordinated behavior, service-oriented robotics

## Abstract

In this paper, we consider the problem of autonomous exploration of unknown environments with single and multiple robots. This is a challenging task, with several potential applications. We propose a simple yet effective approach that combines a behavior-based navigation with an efficient data structure to store previously visited regions. This allows robots to safely navigate, disperse and efficiently explore the environment. A series of experiments performed using a realistic robotic simulator and a real testbed scenario demonstrate that our technique effectively distributes the robots over the environment and allows them to quickly accomplish their mission in large open spaces, narrow cluttered environments, dead-end corridors, as well as rooms with minimum exits.

## 1. Introduction

In recent years, the use of Multi-Robot Systems (MRS) has become popular for several application domains such as exploration, surveillance, and even search and rescue. The main reason for using these MRS is that they are a convenient solution in terms of costs, performance, efficiency, reliability, and reduced human exposure. Most of the time, robots are intended to cooperate so as to accomplish tasks faster than a single robot, to include redundancy and thus robustness, and to compensate sensor uncertainty by combining information [1].

However, the cooperation of MRS has involved additional state-of-the-art problems such as the *coordination* to achieve efficient navigation while avoiding interferences, *resource management* and *information sharing* so as to enhance cognition for task allocation and mapping, and *suitable communication* systems [2]. Research has witnessed a large body of significant advances in the control of single mobile robots, dramatically improving the feasibility and suitability of cooperative approaches, turning the particular domain of efficient exploration of unknown environments a fundamental problem in mobile robotics.

The main goal in robotic exploration is to minimize the overall time for covering an unknown environment. It has been widely accepted that the key for efficient exploration is to carefully assign robots to sequential targets until the environment is covered, the so-called *next-best-view* (NBV) problem [3]. Typically, those targets are called *frontiers*, which are boundaries between open and unknown space that are gathered from range sensors and sophisticated mapping techniques [4].

Allocating those targets among a pool of robots has been addressed from a range of different deliberative perspectives, including economy-based negotiation [5–8], particular costs and utilities [1,2,9–13], learning structures in the environment [11,14], and even by selecting the less compromising targets in terms of sensor errors and communication ranges [15–18]. It is worth mentioning that there are also few reactive exploration approaches that have worked by randomly navigating or even wall-following [19,20], but are less efficient and easily compromised.

Furthermore, highly uncertain and unstructured environments hinder the possibilities for implementing deliberative exploration algorithms, and compromises purely reactive techniques. In addition, these environments make it hard for robots to even keep their footing, thus a good localization and mapping becomes even a bigger challenge. For these cases, literature advises that with no specific strategy or plan but with simple emergence of efficient local behaviors, complex global exploration can be achieved [21]. The algorithm proposed herein is able to deal with this kind of environments.

In this paper, we present an algorithm for single and multi-robot efficient autonomous exploration. Rather than using typical target assignment we focus on gathering new locations by enhancing reactive solutions with a local spatial information memory. The used memory data structure allows for a reduced search complexity of O(1), which combined with a Finite State Automata (FSA) allows us to demonstrate that our technique effectively distributes the robots over the environment and allows them to quickly accomplish their mission. This results in a hybrid approach capable of fast, simple and computational efficient exploration. A series of simulated experiments were performed and analyzed, demonstrating the successful exploration of a 200 m^2^ area in about a minute with a team of three Pioneer-3DX robots at moderate driving and steering speeds and equipped essentially with a laser scanner. Additionally, successful results were demonstrated in real experiments with a team of two Jaguar V2 robots, also equipped with laser scanners, in a 20 m^2^ area geometrically similar to the simulation scenario. One of the most interesting observations is that we achieved qualitatively similar navigation and robot distribution as in the literature but with a way simpler approach, using less computational power, and without any negotiation or mapping technique, at the only cost of sufficient localization so as to handle 1 m^2^ error.

This paper is structured as follows: first, autonomous exploration approaches are detailed in Section 2; next we present our behaviors and FSA design in Section 3; Section 4 follows with the implemented experiments and results; and finally, we present in Section 5 a summary of contributions and conclusions, as well as our future directions.

## 2. Related Work

Many approaches have been proposed for exploring unknown environments with single and multiple robots. A deep comparison on several autonomous exploration approaches has been reported in [22] including their main pros and cons. In most of these, the essence is to allow the robots to autonomously navigate to the best unexplored area. Area representation makes the main difference among different techniques. Nevertheless, a popular basis is the frontier-based exploration algorithm introduced by Yamauchi in [4].

Main advantages of frontier exploration reside in that there is no world structure required, and that it is useful in cluttered environments and acceptable in large open spaces [23]. On the other hand, the necessity for robust localization and mapping impacts directly on algorithm complexity and the need for world representation. Basically, the exploration cycle requires to: gather sensor data, share/merge evidence grids (maps), extract edges and match locations, allocate frontiers, and reliably navigate towards the allocated frontier.

Concerning frontiers' allocation, significant works have been proposed using market-based techniques. In [5] a successful multi-robot exploration is achieved with a bidding process. Nevertheless, the need for a central bid-evaluator agent is an undesired compromise. Moreover, in [7], sequences of potential target locations for each robot are traded between the robots using single-item first-price sealed-bid auctions. Other auction-based techniques have also been applied in [6]. Such methods are likely to show similar performance to the most popular and efficient *utilities* approach.

The utilities approach was introduced by Burgard *et al.* in [9]. The essence is to compute a distance cost towards each frontier and then update the utilities of neighboring frontiers according to greedy assignments of the teammates. This work has been enhanced by using machine learning such as clustering frontiers [11] and learning typical indoor environments [2,14] for more certain mapping and better goal point assignment. The best work in terms of efficient exploration and algorithm complexity reports an O(*n*^2^*T*) for greedy frontier allocation for *n* robots and *T* frontiers, and optimal allocation with up to $\text{O}(\frac{T!}{(T-n)!})$, without considering any additional processing [1].

On the other hand, some works have focused on determining costs that try to minimize the uncertainty of the process. In [16] the idea is to keep the robots in line of sight so that they can help each other to accurately localize while each one navigates in a round-robin process. If robots tend to go out of range, then a rendezvous is scheduled. In [15], the goal is to assign targets that are estimated to reduce the odometry error. Balch and Arkin in [19] propose an anchored random exploration such that every robot remains inside communication range. Nevertheless, even when these works have similar results to the utilities technique and have the advantage of improving the overall accuracy of the map, they have the disadvantage of keeping the robots in close proximity, which can increase the exploration time by preventing the robots from efficiently spreading.

Other works include [8,24] in which they leverage the work in [9] by including communication limits in the cost function and allocating frontiers within a bidding process. Also, in [17], an inside-communication technique demonstrates similar results to the work in [19]. Moreover, with the same rendezvous idea of [16], researchers propose in [18] a role-based technique for allowing robots to explore distant areas while communicating with a *relay* robot so as to return all attained knowledge to a particular location.

The last approach worth to mention is the so-called multi-objective or multi-criteria exploration. In this kind of exploration authors use multiple utility functions so as to allocate locations either among single or multiple robots. In [13], authors focused on the development of strategies by using a multi-criteria decision making process for the robot to decide where to go next. The main idea is to determine the best location by taking into account several criteria such as the open area beyond the target location, the probability of being able to communicate the gathered information once the target has been reached, and the distance from the current position to the target location. Other examples of this kind of exploration are presented in [10,12] in which authors define multiple strategies for exploration in search and rescue missions. The main idea is to determine the best location by considering predefined plans such as *victims first* or *fire first* according to the current scenario. Both ideas have demonstrated interesting results, showing good performance and flexibility for defining multiple exploration strategies. Nevertheless, in [13] it is difficult to define the appropriate weights for each included criteria; while in [10,12] there is the necessity of well characterized scenarios so that predefined plans can apply.

As can be seen, every autonomous exploration approach has its pros and cons. More sophisticated works try to coordinate robots such that they do not tend to move toward the same unknown area while having a balanced target location assignment with less interferences between robots. Furthermore, recent works tend to include communications as well as other strategies for better MRS functionality into the target allocation process. Nevertheless, the reality is that most of these NBV-based approaches still fall short of presenting an MRS that is reliable and efficient in exploring highly uncertain and unstructured environments, robust to robot failures and sensor uncertainty, and effective in exploiting the benefits of using a multi-robot platform.

Hence, the main contribution here is an algorithm that, without explicit target locations and with a much simpler operations cycle, is able to do efficient exploration of unknown environments. Furthermore, we observed territorial exploration (coverage) emergent behavior as a consequence of our exploration approach. It is worth referring that we do not rely on any specific robot so that even at robot failure, mission is not compromised. Additionally, our algorithm has been developed in a service-oriented approach, thus leveraging code re-use, among other important advantages such as the inherent capability of dynamic disconnection/connection for robots that leave the communication ranges [25].

## 3. Methodology

Having presented some relevant works, this section describes our particular approach. We focus on the very basis of robotic exploration according to Yamauchi: “Given what you know about the world, where should you move to gain as much new information as possible?” [4]. In this way, we propose a behavior-based approach for multi-robot exploration that puts together the simplicity and good performance of purely reactive control with some of the benefits of deliberative approaches, regarding the ability of reasoning about the environment.

Besides the careful assignment of targets, we consider other relevant literature advices such as to avoid travelling unnecessary long distances, doing redundant work, interfering with other teammates, wasting time, and depending on individual robots [14]. Also, we consider essential that the robots keep track of which areas of the environment have already been explored [1], concerning about the explicit need of an adequate balance in computational cost, response time and coherence towards the goal. Moreover, our solution addresses issues such as modularity, code reuse, integration and hardware abstraction for which we implemented service-oriented robotics design. A complete description on its benefits can be found at [25].

The proposed solution makes use of four different robotic behaviors and a resultant emergent behavior. It is important to mention that according to pioneer works, one of the essential sensors for an autonomous robot is a laser range finder [26]. This sensor and approximate 1-meter localization are key in our behaviors development. For the simulated and real robots we use a laser sensor with a field of view of 180° and 130° respectively, and a maximum distance of 2 m. In respect to robots' localization, we use the simulation engine to provide us with these values, while for real implementations we use an external vision-based tracking device communicating each robot's pose to a central station to which robots were always able to communicate (direct communication between the robots was disabled). These simplifies our tests by providing a minimal error in localization including a same reference frame for all robots, while enabling us to focus in the particular advantages of our proposed solution. The description of each behavior is presented below.

### 3.1. Avoid Obstacles

The first behavior is the **Avoid Obstacles**. This protective behavior considers 3 particular conditions for maintaining the robot's integrity. The first condition is to check for possible corners in order to avoid getting stuck or spending unnecessary time there because of the avoiding the past effect. The methodology for detecting the corners is to check for the distance measurements of 6 fixed laser points for each side (left, right, front) and according to their values determine if there is a high probability of being a corner. There are multiple cases considering corners: (1) if the corner has been detected at the left, then robot must turn right with an equivalent steering speed according to the angle where the corner has been detected; (2) if it has been detected at the right, then robot must turn left with an equivalent steering speed according to the angle where the corner has been detected; and (3) if the corner has been detected at the front, then robot must turn randomly to right or left with an equivalent steering speed according to the distance towards the corner. The next condition is to keep a safe distance to obstacles, steering away from them if it is still possible to avoid collision, or translating a fixed safe distance if obstacles are already too close. The third and final condition is to avoid teammates so as not to interfere or collide with them. Most of the times this is done by steering away from the robot nearby, but other times we found it useful to translate a fixed distance. It is worth to refer that the main reason for differentiating between teammates and moving obstacles resides in that we can control a teammate so as to make a more efficient avoidance. Pseudocode referring to these operations is presented in Algorithm 1.


**Algorithm 1.** Avoid Obstacles Pseudocode.*AvoidingObstacleAngle* = 0;Check the distance measurements of 18 different laser points (6 for left, 6 for front, and 6 for right) that imply a high probability of *CornerDetected* either in front, left or right;**if**
*CornerDetected*
**then** *AvoidingObstacleAngle* = an orthogonal angle towards the detected corner side;**else** Find nearest obstacle location and distance within laser scanner data; **if**
*Nearest Obstacle Distance* < *Aware of Obstacles Distance*
**then**  **if**
*Nearest Obstacle Distance is too close*
**then**   do a fixed backwards translation to preserve the robot's integrity;  **else**   *AvoidingObstacleAngle* = an orthogonal angle towards the nearest obstacle location;  **end** **else**  **if**
*Any Kins' Distance* < *Aware of Kin Distance*
**then**   With 30% chance, do a fixed translation to preserve the robot's integrity;   With 70% chance, *AvoidingObstacleAngle* = an orthogonal angle towards the nearby kin's location;  **else**   Do nothing;  **end** **end****end****return**
*AvoidingObstacleAngle*;

### 3.2. Avoid Past

The second behavior is for gathering the newest locations: the **Avoid Past**. This kind of explorative behavior was introduced by Balch and Arkin in [27] as a mechanism for avoiding local minima when navigating towards a goal. It was proposed also for autonomous exploration, but it lead to a constant conflict of getting stuck in corners, therefore showing the importance of anticipating and avoiding corners in the previous behavior. Additionally, the algorithm required a static discrete environment grid which must be known at hand, which is not possible for unknown environments. Furthermore, the complexity in order to compute the vector so as to derive the updated potential field goes up to O(*n*^2^) for a supposed *n* × *n* grid world. Thus, the higher the resolution of the world (smaller grid-cell size), the more computational power required. Nevertheless, it is from them and from the experience presented in works such as in [20] that we considered the idea of enhancing reactivity with local spatial memory so as to produce our own algorithm.

**Figure 1.**
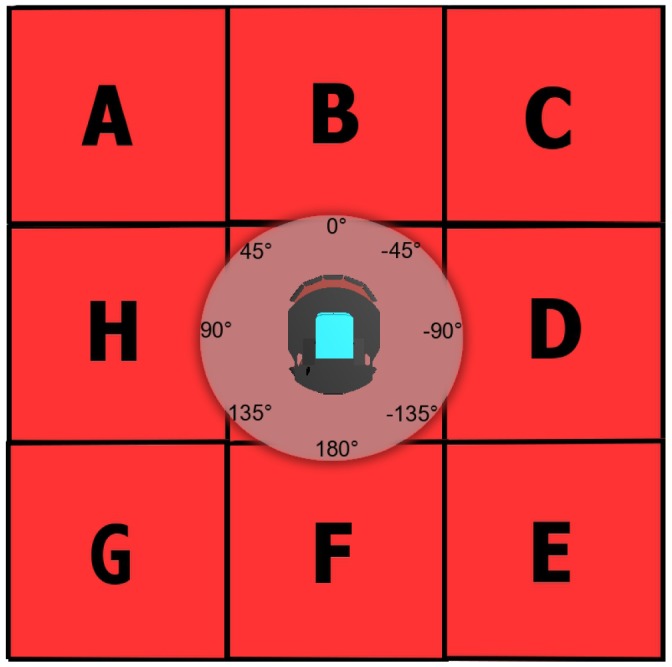
8 possible 45° heading cases with 3 neighbor waypoints to evaluate so as to define a CCW, CW or ZERO angular acceleration command. For example, if heading in the −45° case, the neighbors to evaluate are B, C and D, as left, center and right, respectively.

Our **Avoid Past** does not get the aforementioned problems. First of all, because of the simple recognition of corners provided within the Avoid Obstacles, we never get stuck nor spend unnecessary time there. Next, we are using a *hash table* data structure for storing the robot traversed locations (the past). Basically, concerning the size of the used robots, we consider an implicit 1 m grid discretization in which the actual robot position (*x*,*y*) is rounded. We then use a fixed number of digits, for *x* and *y*, to create the string “*xy*” as a key to the hash table, which is queried and updated whenever the robot visits that location. Thus, each location has a unique key, turning the hash table to be able to look up for an element with complexity O(1), which is a property of this data structure. It is important to mention that this discretization can accommodate imperfect localization within the grid resolution and we do not require any a-priori knowledge of the environment. To set the robot direction, a steering speed reaction is computed by evaluating the number of visits of the 3-front neighbor (*x*,*y*) locations in the hash table. These 3 neighbors depend on the robot orientation according to 8 possible 45° heading cases (ABC, BCD, CDE, DEF, EFG, FGH, GHA, HAB) shown in Figure 1. It is important to notice that evaluating 3 neighbors without a hash table data structure will turn our location search complexity into O(*n*) for *n* locations, where *n* is an increasing number as exploration goes by, thus the hash table is very helpful. Additionally, we keep all operations with the 3 neighbors within IF-THEN conditional checks leveraging simplicity and reduced computational cost. Pseudocode referring to these operations is presented in Algorithm 2.


**Algorithm 2.** Avoid Past Pseudocode.*AvoidingPastAngle* = 0;Evaluate the neighbor waypoints according to current heading angle;**if**
*Neighbor Waypoint at the Center is Free and Unvisited*
**then** *AvoidingPastAngle* = 0;**else** **if**
*Neighbor Waypoint at the Left is Free and Unvisited*
**then**  *AvoidingPastAngle* = 45; **else**  **if**
*Neighbor Waypoint at the Right is Free and Unvisited*
**then**   *AvoidingPastAngle* = −45;  **else**   *AvoidingPastAngle* = an angle between −115 and 115 according to visit counts proportions of the left, center and right neighbor waypoints;  **end** **end****end****return**
*AvoidingPastAngle;*

### 3.3. Locate Open Area

The third behavior, named **Locate Open Area**, is composed of an algorithm for locating the largest open area in which the robot's width fits. It consists of a wandering rate that represents the frequency at which the robot must locate the open area, which is basically the biggest surface without obstacles being perceived by the laser scanner. So, if this behavior is triggered, the robot stops moving and turns towards the open area to continue its navigation. This behavior represents the wandering factor of our exploration algorithm and is very important for the obtained performance. For example, when the robot enters a small room, it tends to be trapped within its past and the corners of the room. If this happens, there is still the chance of locating the exit as the largest open area and escape from this situation in order to continue exploring. Pseudocode referring to these operations is presented in Algorithm 3.


**Algorithm 3.** Locate Open Area Pseudocode.Find the best heading as the middle laser point of a set of consecutive laser points that fit a safe width for the robot to traverse, and have the biggest distance measurements;**if**
*DistanceToBestHeading* > *SafeDistance*
**then** Do a turning action towards the determined best heading;**else** Do nothing;**end**

### 3.4. Disperse

The next operation is our cooperative behavior called Disperse. This behavior is inspired by the work of Matarić [28]. It activates just in the case two or more robots get into a predefined *comfort zone*. Thus, for *m* robots near in a pool of *n* robots, where *m* ≤ *n*, we call for simple conditional checks so as to derive an appropriate dispersion action. It must be stated that this operation serves as the coordination mechanism for efficiently spreading the robots as well as for avoiding teammates interference. Even though it is not active at all times, if (and only if) it is triggered, a temporal O(*m*^2^) complexity is added to the model, which is finally dropped when the *m* involved robots have dispersed. The frequency of activation depends on the number of robots and the relative physical dimensions between robots and the environment, which is important before deployment decisions. Actions concerning this behavior include steering away from the nearest robot if m = 1, or steer away from the centroid of the group of *m* > 1; then a move forward action is triggered until reaching out the defined near area or comfort zone. It is important to clarify that this behavior firstly checks for any possible obstacle avoidance action, and if such action exists the dispersion effect is then overridden until the robot's integrity is ensured. Pseudocode referring to these operations is presented in Algorithm 4.

### 3.5. Explore

**Figure 2.**
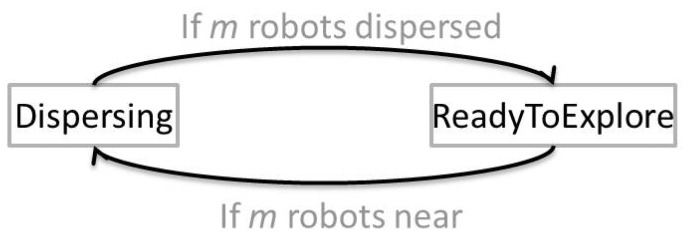
Implemented 2-state Finite State Automata for autonomous exploration.

Last, with a Finite State Automata (FSA) we achieve our Explore emergent behavior. In this emergent behavior, we fuse the outputs of the triggered behaviors with different strategies (either subsumption [29] or weighted summation [27]) according to the current state. In Figure 2 there are 2 states conforming the FSA that results in coordinated autonomous exploration: *Dispersing* and *ReadyToExplore*. Initially, assuming that robots are deployed together, the <*if m robots near*> condition is triggered so that the initial state comes to be *Dispersing*. During this state, the Disperse and Avoid Obstacles behaviors take control of the outputs. As can be appreciated in the Algorithm 4, the Avoid Obstacles behavior overrides (subsumes) any action from the Disperse behavior. This means that if any obstacle is detected, main dispersion actions are suspended. An important thing to mention is that for this particular state, we observed that immediately stopping and turning towards the *AvoidObstacleAngle* (or translating to safety as the Avoid Obstacles behavior commands) was more efficient in order to get all robots dispersed, than by returning a desired angle as the behavior is implemented.


**Algorithm 4.** Disperse Pseudocode.**if**
*Any Avoid Obstacles condition is triggered*
**then** Do the avoiding obstacle turning or translating action immediately (do not return an *AvoidObstacleAngle*, but stop and turn the robot in-situ).; //*Doing this operation immediately and not implementing a fusion with the disperse behavior resulted in a more efficient dispersion effect, this is why it is not treated as the avoid obstacles behavior is implemented*.**else** Determine the number of kins inside the Comfort Zone distance parameter; **if**
*Number of Kins inside Comfort Zone* = = *0*
**then**  **return**
*Status* = *ReadyToExplore*; **else**  *Status* = *Dispersing;*  **if**
*Number of Kins inside Comfort Zone* > *1*
**then**   Determine the centroid of all robots' poses;   **if**
*Distance to Centroid* < *Dead Zone*
**then**    Set *DrivingSpeed equal* to 1.5 * *MaxDrivingSpeed*, and do a turning action to an orthogonal angle towards centroid location;   **else**    Set *DrivingSpeed equal* to *MaxDrivingSpeed*, and do a turning action to an orthogonal angle towards centroid location;   **end**  **else**   **if**
*Distance to Kin* < *Dead Zone*
**then**    Set *DrivingSpeed equal* to 1.5 * *MaxDrivingSpeed*, and do a turning action to an orthogonal angle towards kin location;   **else**    Set *DrivingSpeed equal* to *MaxDrivingSpeed*, and do a turning action to an orthogonal angle towards kin location;   **end**  **end** **end****end**

Then, once all the robots have been dispersed, the <*if m robots dispersed*> condition is triggered so that the new state comes to be the *ReadyToExplore*. In this state, two main actions can happen. First, if the wandering rate is triggered, the Locate Open Area behavior is activated, subsuming any other action out of turning towards the determined best heading if it is appropriate, or holding the current driving and steering speeds, which means to do/change nothing (refer to Algorithm 3). Second, if the wandering rate is not triggered, we fuse outputs from the Avoid Obstacles and Avoid Past behaviors in a weighted summation. This summation requires for a careful balance between behaviors gains for which the most important is to establish an appropriate *AvoidPastGain* < *AvoidObstaclesGain relation* [27]. In this way, with this simple 2-state FSA, we ensure that robots are constantly commanded to spread and explore the environment. Thus, it can be referred that this FSA constitutes the deliberative part in our algorithm since it decides which behaviors are the best according to a given situation, so that the combination of this with the behaviors' outputs lead us into a hybrid solution such as the one presented in [30]. The main difference is that we do not calculate any forces, potential fields, nor have any sequential targets, thus reducing complexity and avoiding typical local minima problems. Pseudocode referring to these operations is presented in Algorithm 5.


**Algorithm 5.** Explore Pseudocode.**if**
*Status* = *Dispersing*
**then** Disperse;**else** **if**
*Wandering Rate triggers*
**then**  *LocateOpenArea*; **else**  Get the current *AvoidingPastAngle* and *AvoidingObstacleAngle*;  //This is to do smoother turning reactions with larger distances towards obstacles;  **if**
*Distance to Nearest Obstacle in Front* < *Aware of Obstacles Distance*
**then**   *DrivingSpeedFactor* = *DistancetoNearestObstacleinFront/AwareofObstacleDistance*;  **else**   *DrivingSpeedFactor* = 0;  **end**  *DrivingSpeed* = *DrivingGain* * *MaxDrivingSpeed* * (1 − *DrivingSpeedFactor*);  //Here is the fusion (weighted summation) for simultaneous obstacles and past avoidance;  *SteeringSpeed* = *SteeringGain* * ((*AvoidingPastAngle* * *AvoidPastGain* + *AvoidingObstacleAngle* * *AvoidObstaclesGain*)/2);  Ensure driving and steering velocities are within max and min possible values;  Set the driving and steering velocities; **end** **if**
*m robots near*
**then**  *Status* = *Dispersing* **end****end**

**Figure 3.**
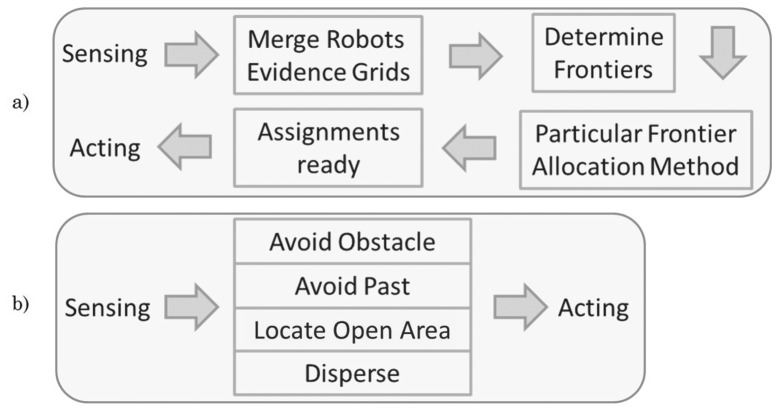
Comparison between: (a) popular literature exploration process and (b) our proposed exploration. Clear steps and complexity reduction can be appreciated between sensing and acting.

Finally, before showing the gathered results, it is important to look at Figure 3 so as to understand the biggest difference between our proposed solution and the popular ones over literature. As can be seen, we require quite fewer steps and computational power since merging the evidence grids, identifying frontiers and allocating them are actions way more complex than our behavioral fusion.

## 4. Experiments

This section describes the experimental details and assumptions, and presents the obtained results. For the ease of understanding it is divided in simulation tests and real implementations for both single and multi robot exploration.

### 4.1. Simulation

For testing purposes, we used a set of Pioneer-3DX robots in their simulated version for Microsoft Robotics Studio, which is a highly realistic simulator as demonstrated in [31]. The simulator provided us with 3D environments and a reliable physics engine, as well as robots' poses. For better appreciation of our results, we implemented a 200 m^2^ 3D simulated environment qualitatively equivalent to the used in Burgard's work [1], one of the most relevant in recent literature. Also, additional environments are included to demonstrate the robustness of the proposed solution across different scenarios such as large open areas, cluttered environments, dead-end corridors, and rooms with minimum exits. Robots are equipped with laser range scanners limited to 2 m and 180° view, and have a maximum driving speed of 0.5 m/s. As for metrics, we used the percentage of explored area over time as well as a exploration quality metric proposed to measure the balance of individual exploration within multiple robots [7], refer to Table 1. Other than these, qualitative appreciation of the robots' traversed paths is presented.

#### 4.1.1. Single Robot Exploration

**Figure 4.**
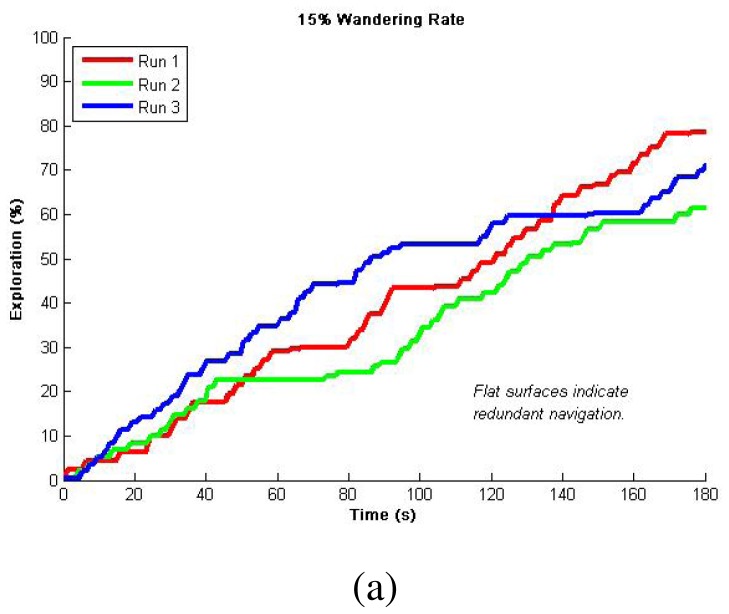

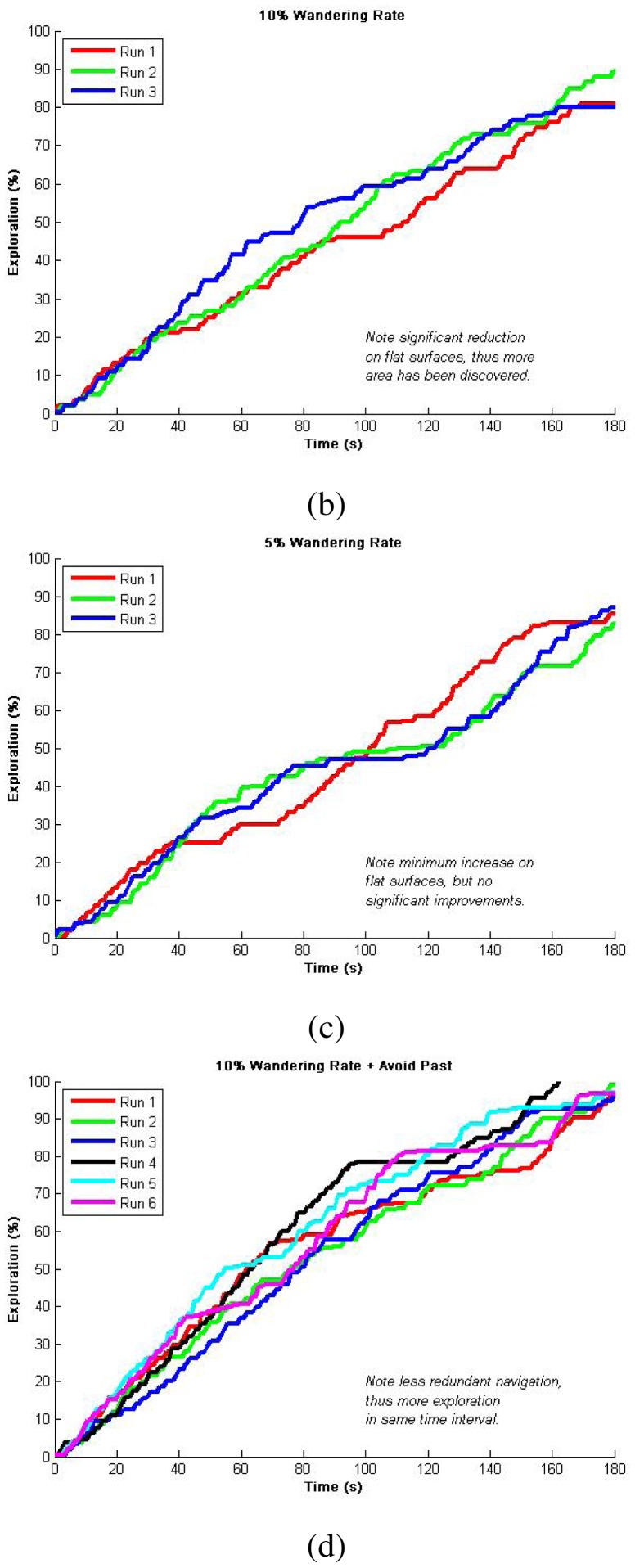
Single robot exploration simulation results: (**a**) 15% wandering rate and flat zones indicating high redundancy; (**b**) Better average results with less redundancy using 10% wandering rate; (**c**) 5% wandering rate shows little improvements and higher redundancy; (**d**) Avoiding the past with 10% wandering rate, resulting in over 96% completion of a 200 m^2^ area exploration for every run using one robot.

**Figure 5.**
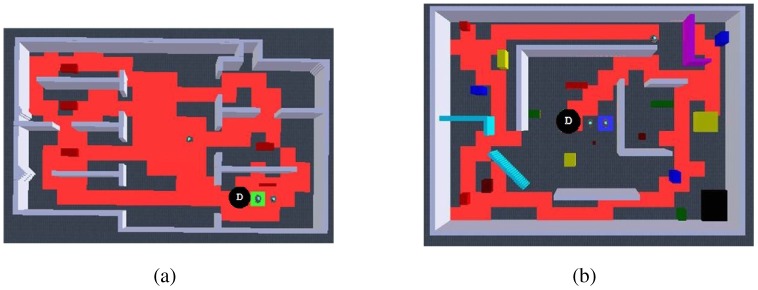
Typical navigation for qualitative appreciation: (**a**) The environment based upon Burgard's work in [1]; (**b**) A second more cluttered environment. Snapshots are taken from the top view and the traversed paths are drawn in red. For both scenarios the robot efficiently traverses the complete area using the same algorithm. Black circle with D indicates deployment point.

Since depending on the robots' proximity our algorithm can either do or not do a dispersion, we decided to test it for an individual robots first. These tests first considered only Avoid Obstacles and Locate-Open-Area operations, known as *Safe Wander*, so as to evaluate the importance of the wandering factor [19]. Figure 4 shows representative results for multiple runs using different wander rates. Since we are plotting the percentage of exploration over time, the flat zones in the curves indicate exploration redundancy (*i.e.*, there was a period of time in which the robot did not reach unexplored areas). Consequently, in these results, we want to minimize the flat zones in the graph so as to refer to a minimum exploration redundancy, while gathering the highest percentage in the shortest time. It is worth to mention that by safe wandering we can not ensure total exploration so we defined a fixed 3 min period to compare achieved explorations. We observed higher redundancy for 15% and 5% wandering rates as presented in Figure 4(a,c), and better results for 10% wandering rate presented in Figure 4(b). This 10% was latter used in combination with Avoid Past to produce over 96% exploration of the simulated area in 3 minutes as can be seen in Figure 4(d). This fusion enhances the wandering so as to ensure total coverage. Statistical analysis from 10 runs is presented in Table 2 for validating repeatability, while typical navigation using this method is presented in Figure 5 as a visual validation of qualitative results. It is important to observe that given the size of the environment and the robot's dimension, one environment is characterized by open spaces and the other provides more cluttered paths. Nevertheless, this very simple algorithm is able to produce reliable and efficient exploration such as more complex counterparts over literature in either open spaces or cluttered environments.

#### 4.1.2. Multi-Robot Exploration

In the literature-based environment, we tested an MRS using 3 robots starting inside the predefined comfort zone (near area) such as in typical robot deployment in unknown environments. First tests considered only Disperse and Safe Wander, which are worth to mention because results sometimes show quite efficient exploration but at other times cannot ensure full exploration. So, this combination may be appropriate in cases where it is preferable to get an initial rough model of the environment and then focus on improving potentially interesting areas with more specific detail (e.g., planetary exploration) [7].

**Figure 6.**
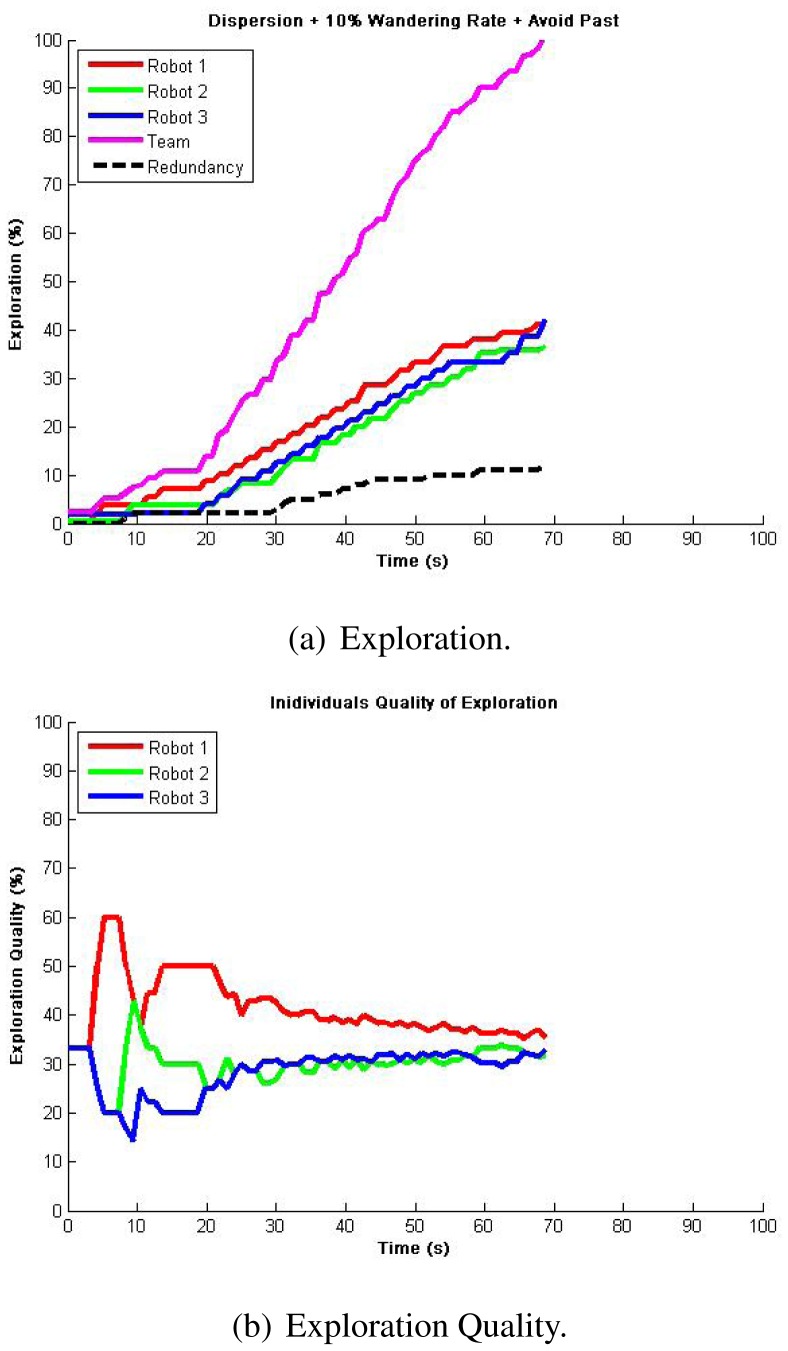
Autonomous exploration showing representative results in a single run for 3 robots avoiding their own past. Full exploration is completed at almost 3 times faster than using a single robot, and the exploration quality shows a balanced result meaning an efficient resources (robots) management.

Nevertheless, more efficient results for cases where guaranteed total coverage is necessary (e.g., surveillance and reconnaissance, land mine detection [32]) were achieved using our exploration algorithm. In our first approach, we intended to be less dependent on communications so that robots avoid their own past only. Figure 6 shows the typical results for a single run with the total exploration on Figure 6(a) and exploration quality on Figure 6(b). We seek for the least flat zones in robots' exploration as well as a reduced team redundancy, which represented locations visited by two or more robots. We can see that for every experiment, full exploration is achieved averaging a time reduction to about 40% of the required time for single robot exploration in the same environment, and even to about 30% without counting the dispersion time. This is highly coherent to what is appreciated in the exploration quality, which showed a trend towards a perfect balance just after dispersion occurred, meaning that with 3 robots we can almost explore 3 times faster. Additionally, team redundancy holds around 10%, representing a good resource management. It must be clear that, because of the wandering factor, not every run gives the same results. However, even when atypical cases occurred, such as when one robot is trapped at dispersion, the team delays exploration while being redundant in their attempt to disperse, then develops a very efficient full exploration in about 50 s after dispersion, which results in a perfectly balanced exploration quality. Table 3 presents the statistical analysis from 10 runs so as to validate repeatability.

**Figure 7.**
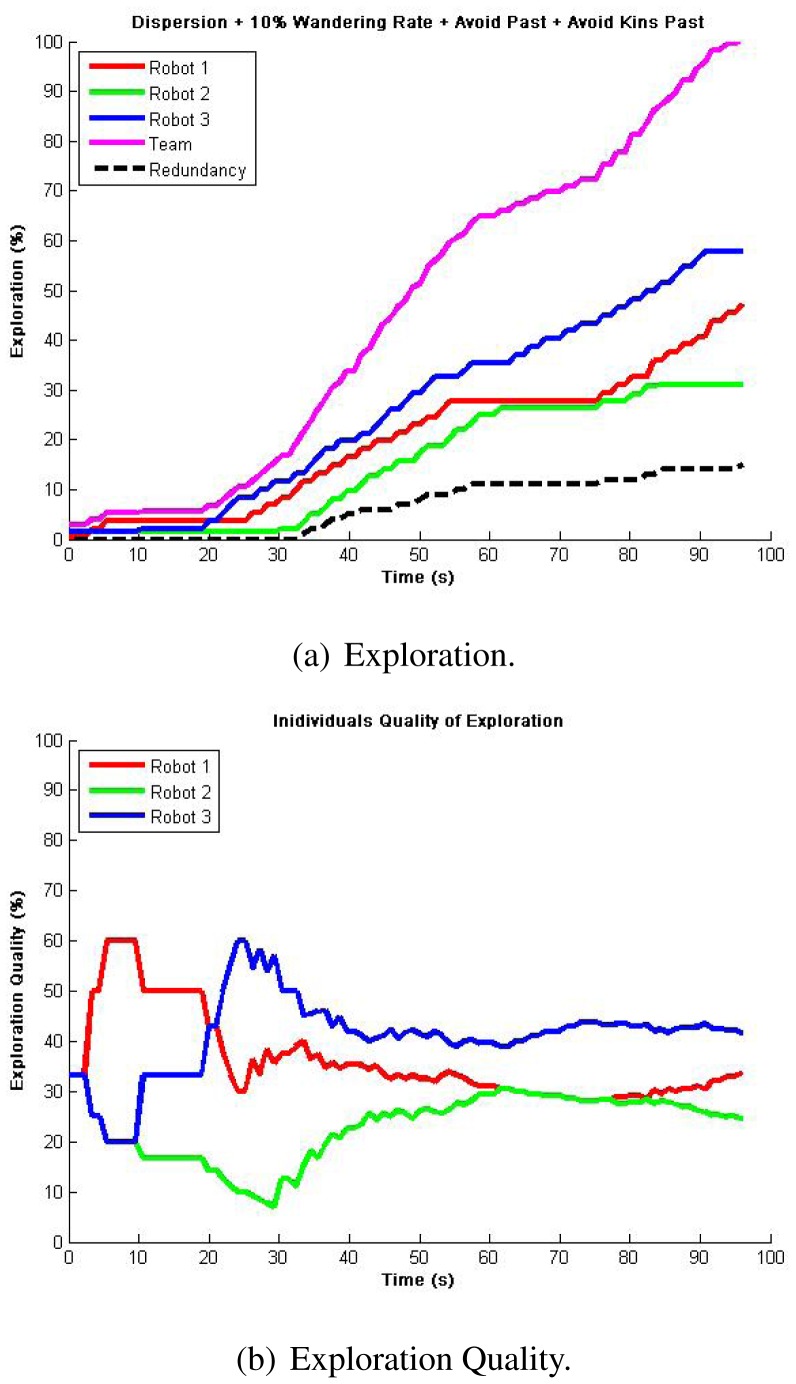
Autonomous exploration showing representative results in a single run for 3 robots avoiding their own and teammates' past. Results show more interference and imbalance at exploration quality when compared to avoiding their own past only.

The next approach consider avoiding also teammates' past. For this case, we assumed that every robot can communicate its location hash table concurrently during exploration, which, as we know, can be a difficult assumption in real implementations. Even though we were expecting a natural reduction in team redundancy, we observed a higher impact of interference and no improvements in redundancy. These virtual paths to be avoided tend to trap the robots, generating higher individuals' redundancy (flat zones) and thus producing an imbalanced exploration quality, which resulted in larger times for full exploration in typical cases, refer to Figures 7(a) and 7(b). In these experiments, atypical cases such as where robots got dispersed the best they can resulted in exploration where individuals have practically just their own past to avoid, thus giving similar results to avoiding their own past only. Table 4 presents the statistical analysis from 10 runs running this algorithm.

**Figure 8.**
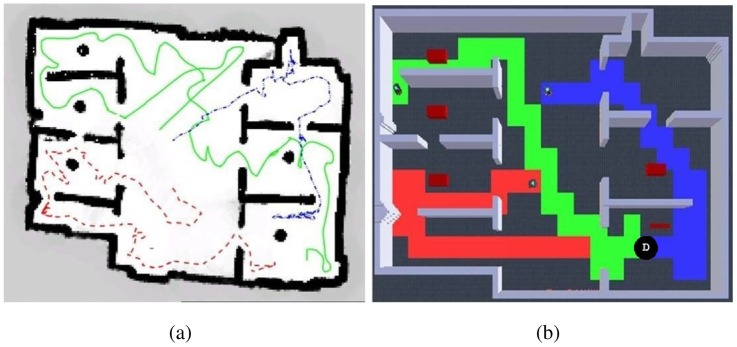
Qualitative appreciation: (**a**) Navigation results from Burgard's work [1]; (**b**) Our gathered results. Path is drawn in red, green and blue for each robot. High similarity with a much simpler algorithm can be appreciated. Black circle with D indicates deployment point.

**Figure 9.**
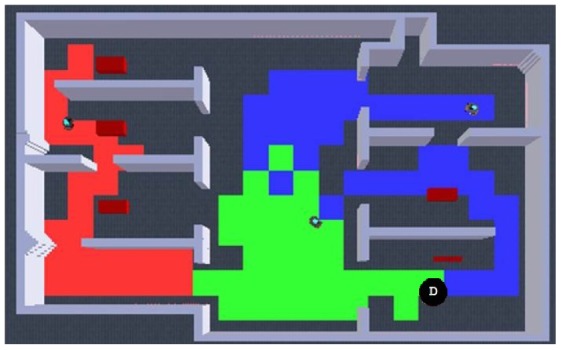
The emergent in-zone coverage behavior for long time running the exploration algorithm. Each color (red, green and blue) shows an area explored by a different robot. Black circle with D indicates deployment point.

Having observed best results by avoiding robots own past and a 10% wandering rate, more experiments were developed. First, in Figure 8 is presented a visual qualitative comparison between Burgard's results and our results. A high similarity can be observed with way different algorithms. Additionally, an interesting observation to exploration results is shown in Figure 9: an emergent behavior that results from running the exploration algorithm for a long time, which can be described as territorial exploration or as in-zone coverage for surveillance tasks [18,32].

**Figure 10.**
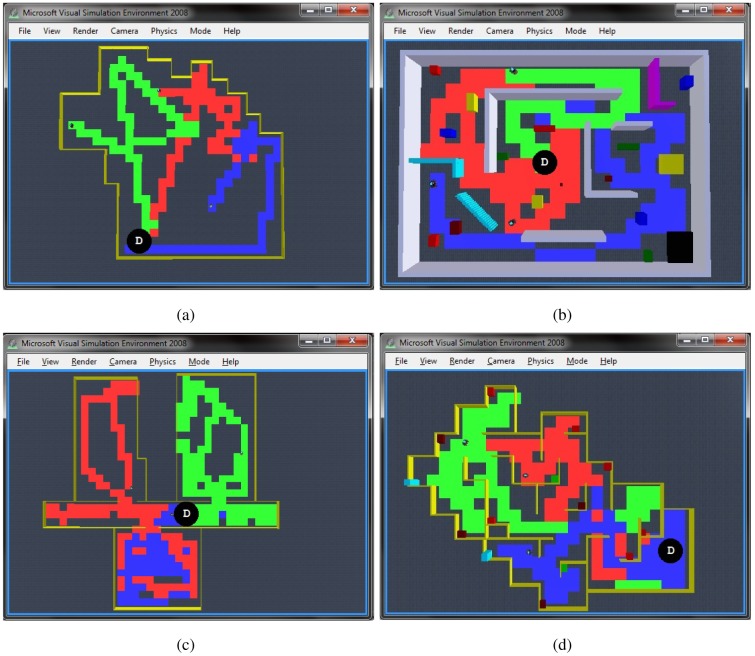
Multi-robot exploration simulation results, appropriate autonomous exploration within different environments including: (**a**) Open Areas; (**b**) Cluttered Environments; (**c**) Dead-end Corridors; (**d**) Minimum Exits. Black circle with D indicates deployment point.

To test the efficacy of the algorithm in different scenarios, in Figure 10 we present the navigation paths of the same autonomous exploration algorithm in different environments including open areas, cluttered areas, dead-end corridors and rooms with minimum exits, all of them with inherent characteristics for challenging efficient exploration. It can be observed that even in adverse scenarios, appropriate autonomous exploration is always achieved. Particularly, we observed that when dealing with large open areas such as in Figure 10(a), robots fulfill a quick overall exploration of the whole environment, but we noticed that it takes more time to achieve an in-zone coverage compared with other scenarios. We found that this could be enhanced by avoiding also kins' past, but it will imply full dependence on communications, which are highly compromised in large areas. Another example is shown in Figure 10(b) considering cluttered environments, these situations demand for more coordination at the dispersion process as well as difficulties for exploring close gaps. Still, it can be observed that robots were successfully distributed and practically achieved full exploration. Next, Figure 10(c) presents an environment that is particularly characterised because of compromising typical potential field solutions because of reaching local minima or even being trapped within avoiding the past and a dead-end corridor. With this experiment we observed that it took more time for the robots to get dispersed and to escape the dead-end corridors in order to explore the rooms, nevertheless full exploration was not compromised and robots successfully navigated autonomously through the complete environment. The final environment shown in Figure 10(d) presents a scenario where the robots were constantly getting inside rooms with minimum exits, thus complicating the efficient dispersion and spreading through the environment. In spite of that, it can be appreciated how the robots efficiently explore the complete environment and achieve in-zone coverage. We observed that the most relevant action for successfully exploring this kind of environments is the dispersion that robots keep on doing each time they face each other.

Summarizing, we have successfully demonstrated that our algorithm works for single and multi-robot autonomous exploration. What is more, we have demonstrated that even being way simpler, it achieves similar results compared with complex solutions in literature. Finally, we have tested its robustness against different scenarios and still get successful results. So, the next step is to demonstrate how it works with real robots.

### 4.2. Real Implementation

Taking advantage from the fast track method towards real implementations described in [31] and from an already working instance of a service-oriented architecture described in [25], experiments with real robots were performed. For the ease of focusing on the performance of our proposed algorithm and taking into account that even the more sophisticated localization algorithms are not good enough for the intended real scenarios, we used an external vision-based device to communicate each robot's pose to a central station, with which robots were always able to communicate. As for the robots, we used a set of two Dr. Robot's Jaguar V2, which are equipped with a laser scanner limited to 2 m and 130° field of view, and a skid-steering mobility platform equivalent to the differential driving of the simulated robots. Maximum driving speed was set to 0.25 m/s, half of the limit in the simulations. The environment consisted in an approximate 1:10 scaled version of the simulation scenario, so that by using the same metrics (refer to Table 1) expected results were available at hand.

#### 4.2.1. Single Robot Exploration

**Figure 11.**
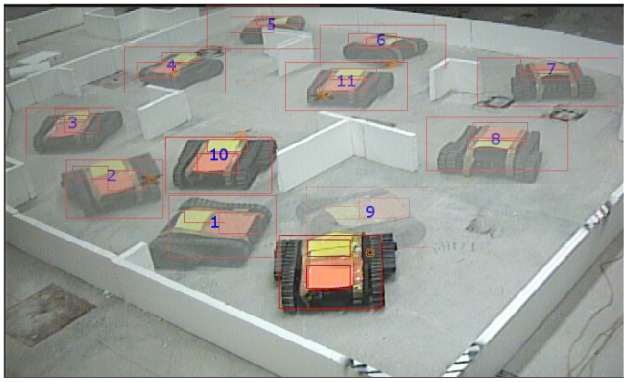
Deployment of a Jaguar V2 for single robot autonomous exploration experiments.

For single robot exploration experiments, a Jaguar V2 was wirelessly connected to an external computer, which was receiving the localization data and human operator commands for starting the autonomous operations (refer to [25] subsystem and system elements). The robot was deployed inside the exploration maze and once the communications link was ready, it started exploring autonomously. Figure 11 shows a screenshot of the robot in the environment, including the tracking and homography markers for localization, and a typical autonomous navigation pattern resulting from our exploration algorithm.

**Figure 12.**
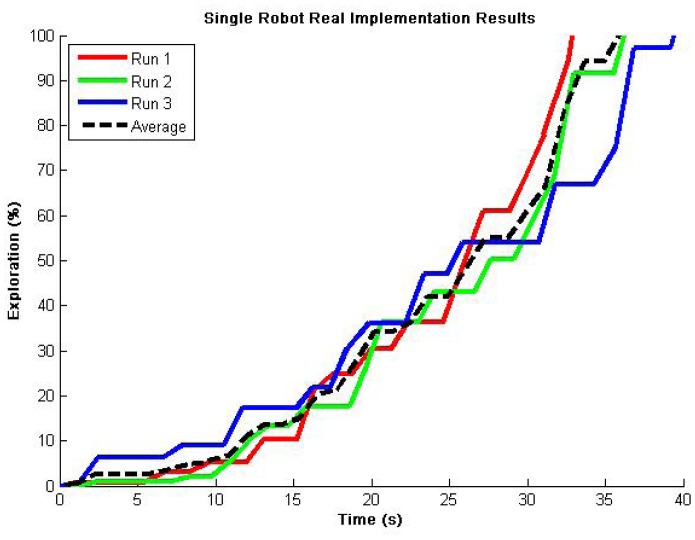
Autonomous exploration showing representative results implementing the exploration algorithm in one Jaguar V2. An average of 36 s for full exploration demonstrates coherent operations considering simulation results.

The maximum speed was set to half the speed of the simulation experiments and the environment area was reduced to approximately 10%. So, the expected results for over 96% explored area must be around 36 s (2 × 180 s / 10 = 36 s, refer to Figure 4(d)). Figure 12 demonstrates coherent results for 3 representative runs, validating our proposed exploration algorithm functionality for single robot operations. There are very few flat zones (redundancy) and close results can be observed among multiple runs, which demonstrates the robustness of the exploration algorithm.

#### 4.2.2. Multi-Robot Exploration

**Figure 13.**
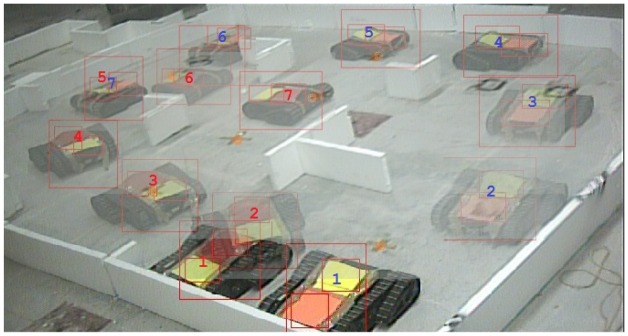
Deployment of two Jaguar V2 robots for multi-robot autonomous exploration experiments.

For the case of multiple robots, a second robot was included as an additional subsystem element as detailed in [25]. Figure 13 shows a screenshot of the typical deployment used during the experiments including the tracking and homography markers for localization, and an example of navigational pattern when the robots meet along the exploration task.

**Figure 14.**
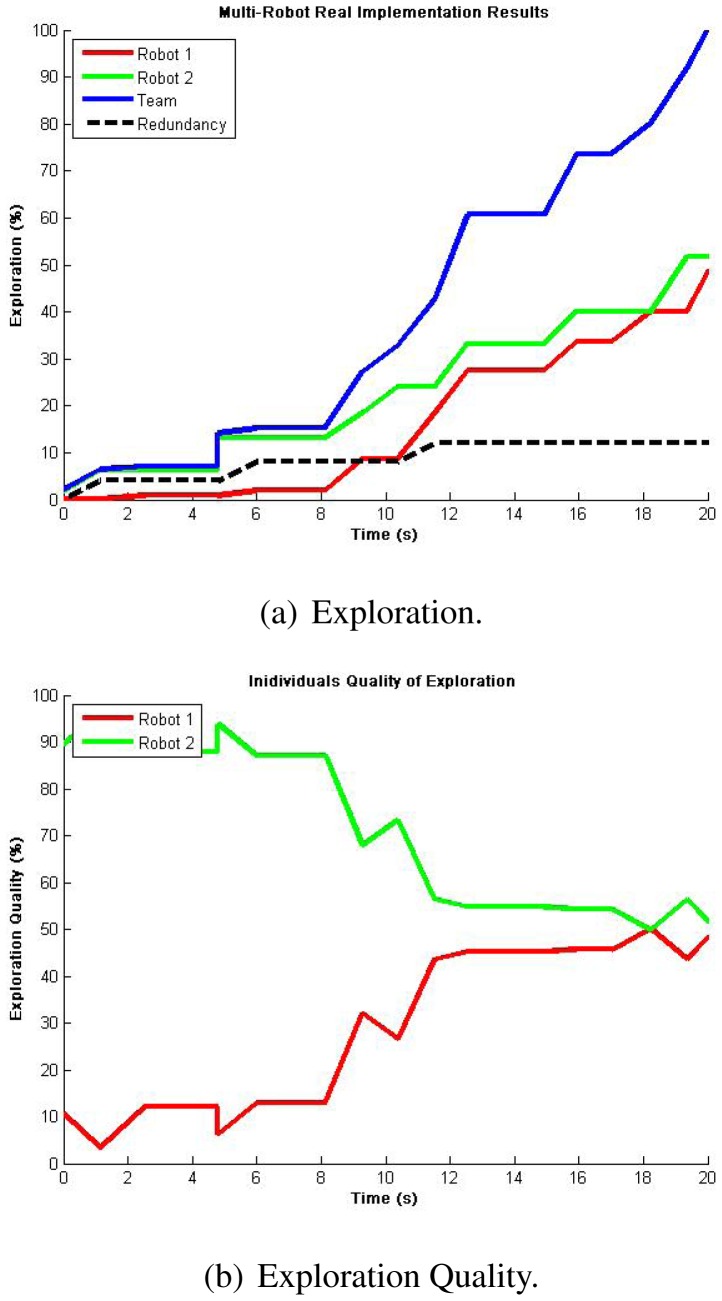
Autonomous exploration showing representative results for a single run using 2 robots avoiding their own past. An almost half of the time for full exploration when compared to single robot runs demonstrates efficient resource management. The resultant exploration quality shows the trend towards perfect balancing between the two robots.

This time, considering the average results from the single robot real experiments, the ideal expected result when using two robots must be around half of the time so as to validate the algorithm functionality. Figure 14(a) shows the results from a representative run including robot's exploration and team's redundancy. It can be appreciated that full exploration is achieved almost at half of the time of using only one robot and that redundancy stays very close to 10%. What is more, Figure 14(b) presents an adequate balance in the exploration quality for each robot. Thus, these results demonstrate the validity of our proposed algorithm when implemented in a team of multiple robots.

## 5. Conclusions and Future Work

We have presented an efficient robotic exploration method using single and multiple robots in 3D simulated environments and in a real testbed scenario. Our approach achieves similar navigational behavior such as most relevant papers in literature including [1,2,11,14,17]. Since there are no standard metrics and benchmarks, it is a little bit difficult to quantitatively compare our approach with others. In spite of that, we can conclude that our approach presented very good results with the advantages of using less computational power, coordinating without any bidding/negotiation process, and without requiring any sophisticated targeting/mapping technique. Furthermore, we differ from similar reactive approaches as [19,20,27], in that we use a reduced complexity algorithm because of hash table data structures with no a-priori knowledge of the environment and without calculating explicit resultant forces or potential fields, thus avoiding any possibility for local minima problems [30,33]. Additionally, we need neither static roles nor relay robots, so that we are free of leaving line-of-sight, and we are not depending on every robot's functionality for task completion. Moreover, we need no specific world structure and no significant deliberation process. In short, our algorithm decreases computational complexity from O(*n*^2^*T*) (*n* robots, *T* frontiers) in deliberative systems and O(*n*^2^) (*n*×*n* grid world) in reactive systems, to O(1) when robots are dispersed and O(*m*^2^) whenever *m* robots need to disperse, and still achieves efficient exploration times, which is largely due to the fact that all operations are composed of simple conditional *if-then* checks and no complex calculations are being done.

Therefore, we have demonstrated that the essence for efficient exploration is to appropriately remember the traversed locations so as to avoid being redundant and time-wasting. Also, by observing efficient robot dispersion and the effect of avoiding teammates past, we demonstrated that interference is a key issue to be avoided. Hence, our critical need is a reliable localization, within the 1 m grid discretization, that can enable the robots to appropriately allocate spatial information. In this way, perhaps a mixed strategy of our algorithm with a periodic target allocation method presented in [15] can give interesting result. What is more, the presented exploration strategy could be extended with additional behaviors that can result in a more flexible and multi-objective autonomous exploration strategy as authors suggest in [13]. The challenge here resides in defining the appropriate weights for each action so that the emergent behavior performs efficiently.

Now, we are intending to include the *service-oriented* property of dynamic discoverability so as to enhance far reaches exploration [18] by allowing the individual robots to connect and disconnect automatically according to communication ranges and dynamically defined rendezvous/aggregation points as in [16]. With this approach, we expect individual robots to leave communications range for a certain time and then autonomously come back to connection with more data from the far reaches in the unknown environment. These following steps include testing in different real environments so as to evaluate the algorithm's robustness. Also, we are looking forward to dispose of the vision-based localization external device so as to give more precise quantitative evaluations such as map quality/utility as referred in [34,35], as well as for enabling the robots to navigate in larger environments. In fact, we are considering the possibility for replicating major literature contributions so as to obtain quantitative evaluations that can enable us to recognize the real benefits that our proposed strategy provides against the more complex coordination techniques. We believe that this can also promote metrics standardization and lead to more comparable contributions.

Furthermore, the long-term goal is to contribute to search and rescue missions, where time constraints generally require to privilege the amount of explored area over the map quality, for which we think there is still a long way in terms of mobility, uncertainty and 3D locations management. Nevertheless, we believe it is by providing these alternative approaches that we can have a good resource for metrics and evaluation purposes that will lead us to address complex problems and effectively resolve them the way they are.

## Figures and Tables

**Table 1.** Metrics used in the experiments.

METRIC	DESCRIPTION	EXAMPLE
EXPLORATION (%)	For single and multiple robots, measures the percent of gathered locations from the total 1-meter grid discrete environment. With this metric we know the total explored area in a given time and the speed of exploration.	In Figure 12, an average of 100% Exploration was achieved in 36 s.
EXPLORATION QUALITY (%)	For multiple robots only, measures how much of the total team's exploration has been contributed by each teammate. With this metric we know our performance in terms of resource management and robot utilization.	In Figure 14(b), two robots reached 100% Exploration with approximately 50% Exploration Quality each.

**Table 2.** Average and Standard Deviation for full exploration time in 10 runs using Avoid Past + 10% wandering rate with 1 robot.

RUNS	AVERAGE	STD. DEVIATION
10	177.33 s	6.8 s

**Table 3.** Average and Standard Deviation for full exploration time in 10 runs using Avoid Past + 10% wandering rate with 3 robots.

RUNS	AVERAGE	STD. DEVIATION
10	74.88 s	5.3 s

**Table 4.** Average and Standard Deviation for full exploration time in 10 runs using Avoid Kins Past + 10% wandering rate with 3 robots.

RUNS	AVERAGE	STD. DEVIATION
10	92.71 s	4.06 s
